# Optimal management of health care for persons with disability related to spinal cord injury: learning from the Sunnaas model of telerehabilitation

**DOI:** 10.1038/s41394-020-00338-6

**Published:** 2020-09-24

**Authors:** Ingebjørg Irgens, Bodil Bach, Tiina Rekand, Sveinung Tornås

**Affiliations:** 1 Sunnaas Rehabilitation Hospital, Bjørnemyrveien 11, 1450 Nesoddtangen, Norway; 2 Institute of Clinical Medicine, University of Oslo, PO Box 1171, Blindern, 0318 Oslo, Norway; 3 SMARTsam AS, Bølgenveien 8, 3514 Hønefoss, Norway; 4 Department of Neurology/Spinal Cord Unit, Haukeland University Hospital, Jonas Lies vei 65, 5053 Bergen, Norway; 5 Sahlgrenska Academy and Institute for Neuroscience and Physiology, University of Gothenburg, Box 100, S-405 30 Gothenburg, Sweden

**Keywords:** Rehabilitation, Health care

Telemedicine [1] has changed the way of offering medical services around the world. It has rapidly been brought to the forefront because of the Covid-19 pandemic [2]. We believe this should be a permanent change and that telemedicine should be included as part of every hospital system of care. Individuals with disabilities, like spinal cord injury (SCI), or persons living far away from specialized care centers often have problems with traveling long distances [3, 4]. Varying weather conditions due to climate change, as well as pandemics with the need to reduce the risk of infection [5] have induced the necessity for health care providers think creatively about seeing patients, rather than patients only have the option to travel to hospitals and outpatient clinics. Telemedicine is a way to overcome these limitations [1–3].

Almost 100 years ago, teleradiology as communication support for the health care providers, was in use on the Queen Mary [6], but it is just recently that telemedicine for medical purposes has expanded all over the world [7]. Today, many options are available for patients in need of long-term follow-up [8]. The purpose of this perspective is to explain how we managed to implement telerehabilitation as part of our system of care, in hopes that others will follow suit.

**Fig. 1:**
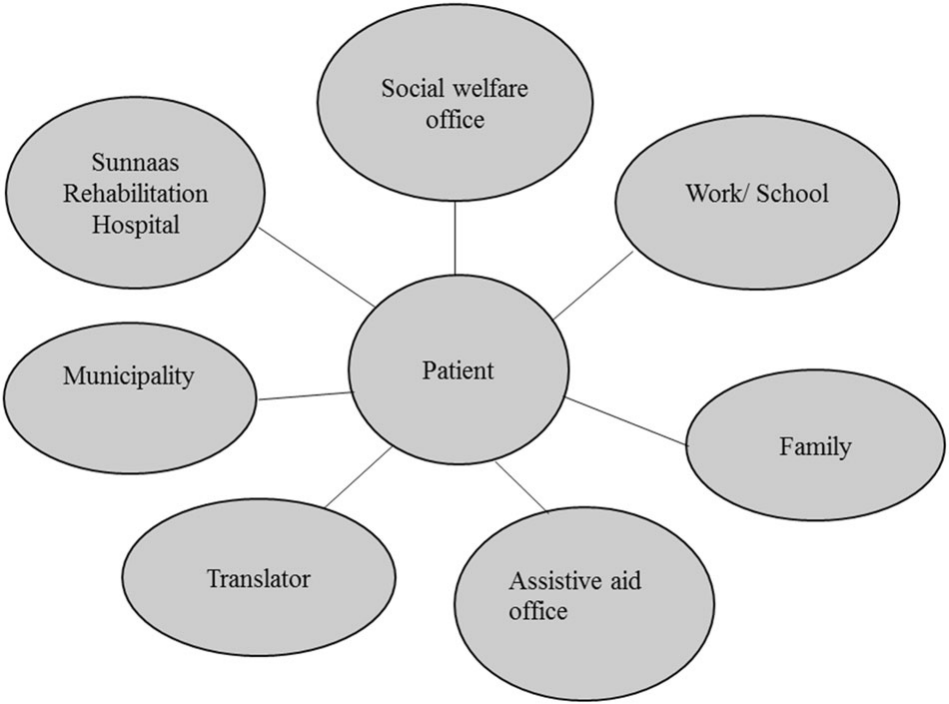
The different participants in the rehabilitation process. To ensure the best possible services to the care receivers during the rehabilitation process, coordination and collaboration from a vast number of collaborators are required.

Sunnaas rehabilitation hospital provides services for individuals in need of highly specialized rehabilitation due to severe impairments, like SCI, multi trauma, burn injury, neurodegenerative conditions, stroke, traumatic brain injury, cerebral palsy, and poliomyelitis. The Spinal Cord Unit at the hospital offers life-long follow-up rehabilitation for individuals with SCI and associated conditions. An important task is to ensure the best possible services to our patients and their families [9, 10], which requires cooperation from a vast number of disciplines (see Fig. 1).

Due to our large catchment area and the geography of Norway, long-term and life-long follow-up are challenging. As a result, we have fostered a successful model of telerehabilitation via videoconference in our health care organization [11–15], the Sunnaas model of telerehabilitation. We believe this model improves public health, and supports more sustainable health services, including accessibility, prevention, earlier treatment and better interaction and knowledge transfer between health care providers on different levels (Fig. 1). Moreover, it is particularly important to ensure good coordination when the responsibility for the patient is transferred between hospitals and municipalities, and between departments and units within hospitals and municipalities [16]. The benefit of including patients, relatives, as well as health care collaborators in treatment team meetings and group decisions is obvious, in particular when this collaborating also protects the environment as there is significantly less driving involved.

This perspective gives a suggestion of how to implement telerehabilitation in the health care service of a hospital.

We define telerehabilitation as communication by videoconferencing via PC, laptop, tablets, or mobile phone to improve the wellness or rehabilitation status of an individual. We have used both integrated and external webcams. Encrypted communication takes place in real time without recording or archiving and must be in accordance with legislation regarding data safety, privacy, and confidentiality applicable in the country/region.

Notes, pictures, and evaluations are documented in the electronic medical record.

Only necessary members of the multidisciplinary team and external care providers participate in the videoconference, and participants must receive training in ethical guidelines and in the use of the equipment before participating as a telerehabilitation provider.

Moreover, despite the fact that we implemented telerehabilitation services 7 years ago into our outpatient system of care for people with SCI [12], we recently expanded this service into other diagnostic groups, such as stroke and cerebral palsy [13, 14]. We have also incorporated exercise supervision via telerehabilitation [17].

Our organizational model is presented in Table 1 (see Table 1). The model includes education and mentoring to staff members, colleagues at collaborative hospitals, local home care providers, patients and their family members. People in need of continuous follow-up, such as individuals with SCI and other severe disabilities, benefit from our approach, which includes local providers, because rehabilitation is an ongoing process and a large part of it takes place after discharge, in the local environment. Thus providing services at home is conducive to optimizing the lifestyle of the person with the disability [8].

**Table 1:** The Sunnaas model of telerehabilitation.

Health service delivery	Intention
Collaborative meeting with the municipality	Multidisciplinary meetings • Before discharge • Before admission • After hospitalization Courses and knowledge exchange
Knowledge translation, meetings, and courses	Courses, competence exchange, and discussions related to specific topics via videoconference, web or e-learning courses. • More participants and more discussion =increased knowledge translation • Learning and coping courses
Assistive aid dissemination	Dialogue with the assistive aid office Increased consumer participation
Interpreter services	Interpreter assisting via videoconferencing • Regional interpreting center is established • Qualified health interpreters • Aim; 40% videoconference interpretation • Great potential also in the municipality
Isolation rooms due to infectious disease	• Patient education • Education about infection routines • Municipal meetings • Interpreter assistance • Dialogue with the nurse-staff room when needed • Fill out forms with help from members of the multidisciplinary team
Consultations with specialists in other hospitals	Patient consultations with specialists in other hospitals Example: • Pressure injury, burn injury, fractures, and spinal cord injury • External camera is connected to the screen to secure detailed visual information
Outpatient follow-up consultations	Consultation with the outpatient clinic • Videoconference to the patient in his and her home • Local health care providers attending • GP attending • Attendance from external medical specialists
Exercise	Physiotherapists performing adjusted exercise via videoconference

Services delivered via videoconference by the multidisciplinary team.

Our inpatient-based service has been especially useful during the Covid-19 pandemic in order to allow people to participate in rehabilitation even if they are in isolation. The model also makes it possible to collaborate with the local care providers while the individual is still an inpatient and thus effectively plan discharge to their municipality. This is particularly important for individuals with life-long follow-up needs, like SCI. Finally, performing videoconference meetings before admittance to the rehabilitation hospital makes it possible to personalize and customize the hospitalization stay.

Rehabilitation services should be available for all individuals with impaired function due to injury or sickness. Thus, health care should be organized and planned to include the provision of rehabilitation services, no matter the geographical location of the caregiver or care receiver. Videoconferencing is a good tool for cooperation in the rehabilitation process [1, 3, 8] and we have found our model is beneficial for all persons who are discharged to home from inpatient rehabilitation. Optimizing the cooperation with local authorities, consumer organizations, and other relevant partners ensures sufficient and adequate capacity, reasonable structure, appropriate expertise and activities in a way that secures quality care and patient safety [9, 10]. The model contributes to effective, safe, and predictable interactions that allow specialized rehabilitation providers to share and transfer their competence to providers at a local level while also including consumers as active participants in the process [11, 12]. It allows the health care system to coordinate follow-up at a local level [18–20] and we believe now more than ever it is important to offer proper treatment at the right place at the right time [16].

Geographical locations can be a barrier to receive the needed rehabilitation service, because long-distance traveling can cause suffering for patients, e.g., patients with SCI and pressure injuries [21]. Expenses associated with transportation are large in terms of time and money, but the expenses are large also in terms of the carbon footprint [21]. Videoconferencing ensures consumers receive necessary follow-up, and makes it possible for caregivers and local health care providers to interact virtually [20, 22]. The end result is proximity at a distance between all participants [11, 19]. Active feedback from consumers, their relatives and the municipal health care service have revealed our telerehabilitation services are more adapted to everyday life [11] for individuals with complex needs in need of long-term follow-up, such as people with SCI [8, 11–15, 18–21].

Having full support of organizational management, technical assistance, and development of dynamic guidelines are important factors for success. Complex management structures, lack of infrastructure, poor communications technology, and founding are barriers to implementation.

The Covid-19 pandemic has resulted in a massive increase in telemedicine services in conjunction with improved funding in some countries. Fortunately, new software-based videoconferencing services, which have recently been available around the world, have decreased the need for start-up costs. However, coordination of care remains problematic and potential solutions need to be addressed. We believe it is important that stakeholders are involved in service development and evaluation and we have done this in the Sunnas model [22].

**Fig. 2:**
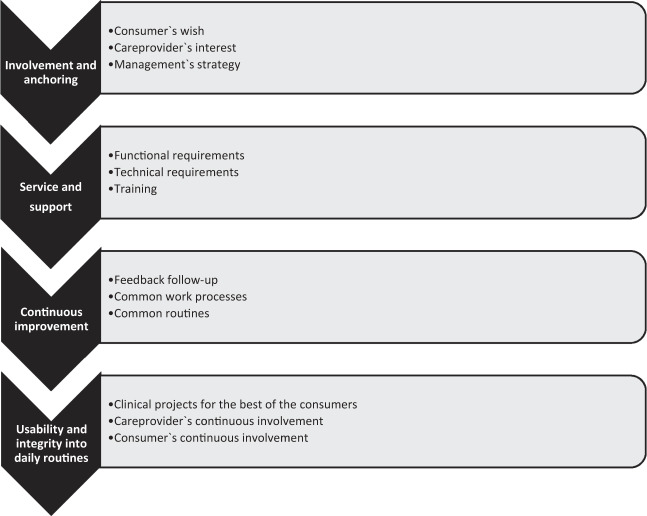
Success criteria in the implementation and usage of new, technological services. The organization must pay attention to user involvement, potential barriers, complexities and context regarding the new solutions.

**Fig. 3:**
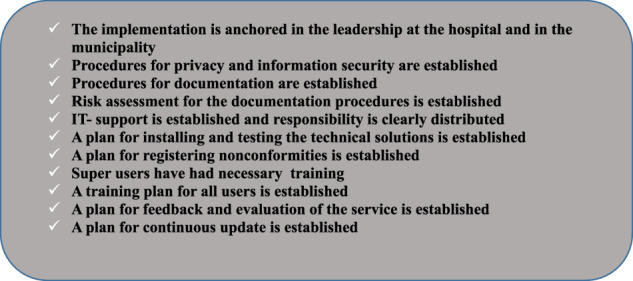
A checklist for development and use of new, technological services. The checklist should focus on ethical issues, availability, professional quality, safety and training for all participants of the service.

Historically, we performed a feasibility study, which lead to implementation of this service in the outpatient clinic [12]. Furthermore, we developed a telemedicine team (TMT) with specialized expertise and dedicated time to be included in all new clinical projects and feasibility studies. Guidelines were developed and were continuously updated and available on the organization’s web page. Figure 2 shows criteria to be taken into account in the implementation of new services (see Fig. 2). Training for all participants of the service remains important as are instructions and check lists (see Fig. 3) with a focus on ethical issues, professional quality, and safety for the participants.

Establishment of an equipment replacement and software update plan and a videoconference-network for sharing experiences and ideas with organizations outside the hospital is important. The success of establishing a telerehabilitation system of care 20% dependent on technology and 80% on organizational support [12, 19, 23, 24].

Our model has been a success in the outpatient follow-up of persons with SCI, facilitating testing and implementation of new, technological health care solutions, and we recommend replication to other patient groups and diagnoses, because the benefits of telerehabilitation are undeniable.
